# Case Report: jaundice in the young: the complexity of rare diseases beyond cholangiopathies

**DOI:** 10.3389/fgstr.2025.1579928

**Published:** 2025-05-09

**Authors:** Simone Guglielmo, Francesca Pasin, Marta Biolo, Claudia Mescoli, Stefania Vio, Luca Fabris, Paolo Simioni

**Affiliations:** ^1^ Department of Medicine (DIMED), University of Padua, Padua, Italy; ^2^ Clinical Medicine 1, Thrombotic and Hemorrhagic Disease Unit, and Hemophilia Center, Padua University-Hospital, Padua, Italy; ^3^ Pathology Unit, Padua University-Hospital, Padua, Italy; ^4^ Radiology Unit II, Padua University-Hospital, Azienda Ospedaliera, Padua, Italy; ^5^ Department of Internal Medicine, Digestive Disease Section, Liver Center, Yale University, New Haven, CT, United States

**Keywords:** autoimmune pancreatitis, inflammatory bowel disease, autoimmune hemolytic anemia, magnetic resonance imaging (MRI), colon biopsy

## Abstract

Jaundice is a common presentation of diseases of the biliary tree, which differential diagnosis can be challenging, in particular when associated to pancreas involvement. In this respect, autoimmune pancreatitis (AIP), a rare form of chronic pancreatitis, shares clinical presentations with pancreatic cancer, such as biliary obstruction and pancreatic mass. AIP is categorized into two subtypes, type 1, associated with elevated serum IgG4 levels and systemic involvement, including the biliary system, and type 2, which is not linked to IgG4 and can be associated with inflammatory bowel disease (IBD). Early recognition is critical as both subtypes respond well to corticosteroid therapy, potentially avoiding unnecessary surgical interventions. Here we discuss the case of a 29-year-old African man with no significant medical history, who presented with skin-scleral jaundice, pale stools, dark urine, and mild weight loss. Laboratory results showed elevated liver and pancreatic enzymes, and imaging revealed bile duct dilation and pancreatic enlargement, raising suspicion of AIP. Serum IgG4 levels were normal, and fecal calprotectin was elevated, suggesting possible IBD. Corticosteroid therapy was initiated, leading to rapid remission of jaundice. One year later, the patient developed gastrointestinal symptoms, mostly abdominal pain and diarrhea, which led to the endoscopic diagnosis of Crohn’s disease. This association further supported the diagnosis of type 2 AIP. The patient subsequently developed a recurrent jaundice due to autoimmune hemolytic anemia (AHIA), a very rare complication of AIP, supported by a positive Coombs test. Once again, corticosteroids resulted in a complete clinical response. This case illustrates the diagnostic challenges of jaundice caused by pancreato-biliary diseases and the wide range of related immunological disorders, i.e. IBD and AHIA, which may influence the clinical presentation. Prompt recognition of the disease enables us to start timely corticosteroid therapy, which confirmed the diagnosis avoiding unnecessary surgery.

## Introduction

In the clinical management of jaundice affecting the young adult, cholangiopathies represent a frequent cause, but the diagnostic work-up is often intricate and time-consuming. A multifaceted approach with careful consideration of the pancreas involvement is essential, as pancreas disease can be associated with primary cholangiopathies, in particular in immune-mediated origin. In this setting, autoimmune pancreatitis (AIP) is an increasingly recognized form of chronic pancreatitis, which can mimic pancreatic cancer in clinical onset, by presenting as pancreatic mass complicated by biliary obstruction. A timely diagnosis is crucial, as prompt corticosteroid treatment may induce the complete clinical remission without biliary stenting. AIP is classified into two subtypes. Type 1 AIP, also known as lymphoplasmacytic sclerosing pancreatitis, is part of the IgG4-related systemic disease, often involving the bile duct system as well as other organs, in particular kidneys and salivary glands, mainly affecting people older than 60-year-old. This subtype is characterized by elevated serum IgG4 levels, which help in distinguishing it from other pancreatic and biliary diseases. In contrast, type 2 AIP, or idiopathic duct-centric pancreatitis, much rarer than type 1 AIP, is not associated with increased serum IgG4 levels and typically affects younger individuals. It is more localized to the pancreas, often in association with inflammatory bowel disease (IBD), i.e. Crohn’s disease or ulcerative colitis. Unlike type 1, organ involvement, in particular bile ducts, is rarely found in type 2 AIP.

Early identification and discrimination of type 1 and type 2 AIP are key steps in the clinical approach to jaundiced patient, since either respond well to corticosteroid therapy, avoiding unnecessary and highly life quality impacting surgical interventions, particularly when AIP mimic pancreatic malignancy. Herein, we discussed a case of type 2 AIP, who presented with obstructive jaundice with later development of associate immune-mediated conditions, including Crohn’s disease and hemolytic anemia.

## Case description

A 29-year-old African man, with unremarkable medical history, presented to the emergency department of the University-Hospital in Padua with complaints of pale stools and dark urine for 7 days, accompanied by scleral jaundice for the past two days. He also reported mild nausea and a 4 kg weight loss over the last three months. The patient denied any recent travel, consumption of spoiled food, alcohol, drugs or fever episodes.

Initial blood tests showed abnormal liver parameters: total bilirubin of 89.6 µmol/L (reference range 1.7 – 17 µmol/L), direct bilirubin of 72.4 µmol/L (reference range 0 – 5.1 µmol/L), aspartate aminotransferase (AST) of 106 U/L (reference range 10 – 45 U/L), alanine aminotransferase (ALT) of 229 U/L (reference range 10 – 50 U/L), gamma-glutamyl transpeptidase (GGT) of 525 U/L (reference range 3 – 65 U/L), and alkaline phosphatase (ALP) of 322 U/L (reference range 43 – 115 U/L). Pancreatic enzymes were concomitantly elevated, though only at a mild degree: alpha-amylase of 109 U/L (reference range 13 – 53 U/L) and pancreatic lipase of 150 U/L (reference range 0 – 60 U/L). Other tests, including blood cell count, renal function, and electrolytes, were normal.

**Figure 1:**
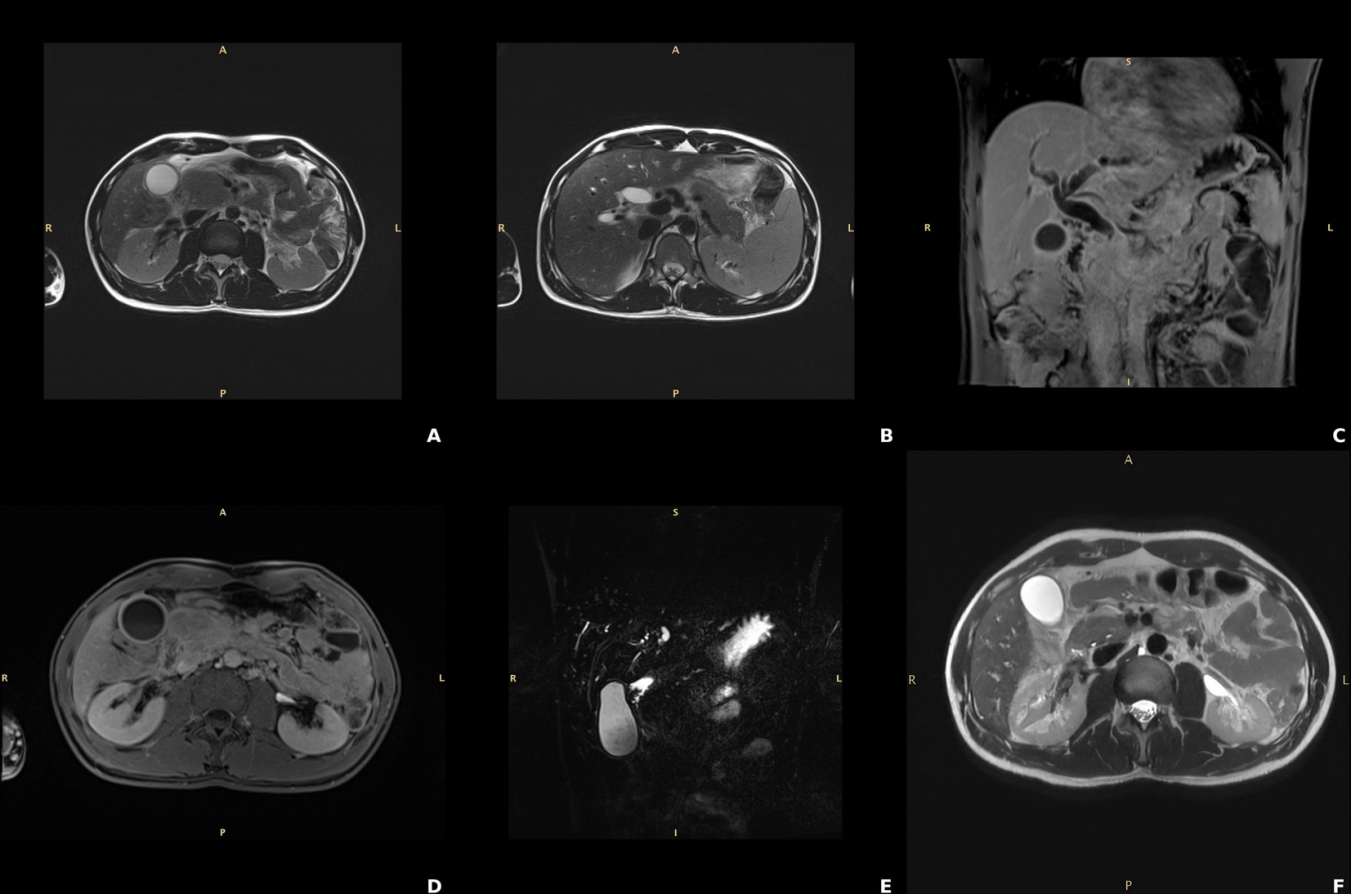
Findings of the contrast-enhanced magnetic resonance of the abdomen consistent with autoimmune pancreatitis. **(A, B)** T2w TSE sequences, axial planes: “sausage like” enlargement of the pancreas particularly expressed at the head. **(C, D)** T1w post contrast VIBE sequences respectively coronal and axial planes showing the post contrast enhancement of the borders of the pancreas (ribbon sign) due to lymphocytic infiltration. **(E)** Cholangiopancreatography, coronal MIP: absent visualization of the main pancreatic duct at pancreatic head which distally is slightly tortuous and not significantly dilatated (penetrating duct sign). **(F)** T2w TSE: normal appearance and decreased size of the pancreatic head compared to the pancreas at diagnosis; biliary and pancreatic duct normal patency.

An abdominal ultrasound revealed marked dilation of the bile ducts, sustained by a stenosis of the intrapancreatic tract of the common bile duct with an enlarged pancreatic head. Further investigations showed normal IgG4 levels (0.148 g/L, reference range 0 – 0.150 g/L), mild positivity for anti-nuclear antibodies (ANA) at a titer of 1:80, with an AC-4 pattern, negativity of the extractable nuclear antigen (ENA) antibodies, high fecal calprotectin (507 µg/g, reference range < 50 ug/g), and significantly low levels of fecal elastase (64 µg/g, reference range > 200 ug/g). Marked bile duct dilation was confirmed by the contrast-enhanced magnetic resonance imaging (MRI) of the upper abdomen, sustained by an enlarged pancreas characterized by a sausage-like appearance within a peripancreatic rim, accompanied by the ‘duct-penetrating sign’, highly suggestive for AIP (**Figures 1A-E**). As jaundice rapidly worsened (total bilirubin to 192.2 µmol/L and direct bilirubin to 158.6 µmol/L after only 2 days), with alpha-amylase rising up to 219 U/L, and lipase to 329 U/L in the same time frame, we decided to start corticosteroid therapy immediately (i.v. 0.75 mg/kg/day), leading to a rapid clinical and biochemical improvement of cholestasis after 3 days (total bilirubin 70.5 µmol/L, direct bilirubin 51.6 µmol/L, GGT 250 U/L, ALP 239 U/L) and normalization of calprotectin levels (16 ug/g). An endoscopic examination aimed at addressing the suspect of IBD was proposed to the patient, who however rejected it due to the absence of colitis-related symptoms. Based on MRI findings, normal IgG4 levels, possible concomitant IBD and biochemical response to steroids, a likely diagnosis of type 2 AIP was made and the patient started chronic prednisone therapy from 60 mg/day with a tapering of 5 mg every 15 days up to the final dosage of 40 mg/day at the discharge.

**Figure 2:**
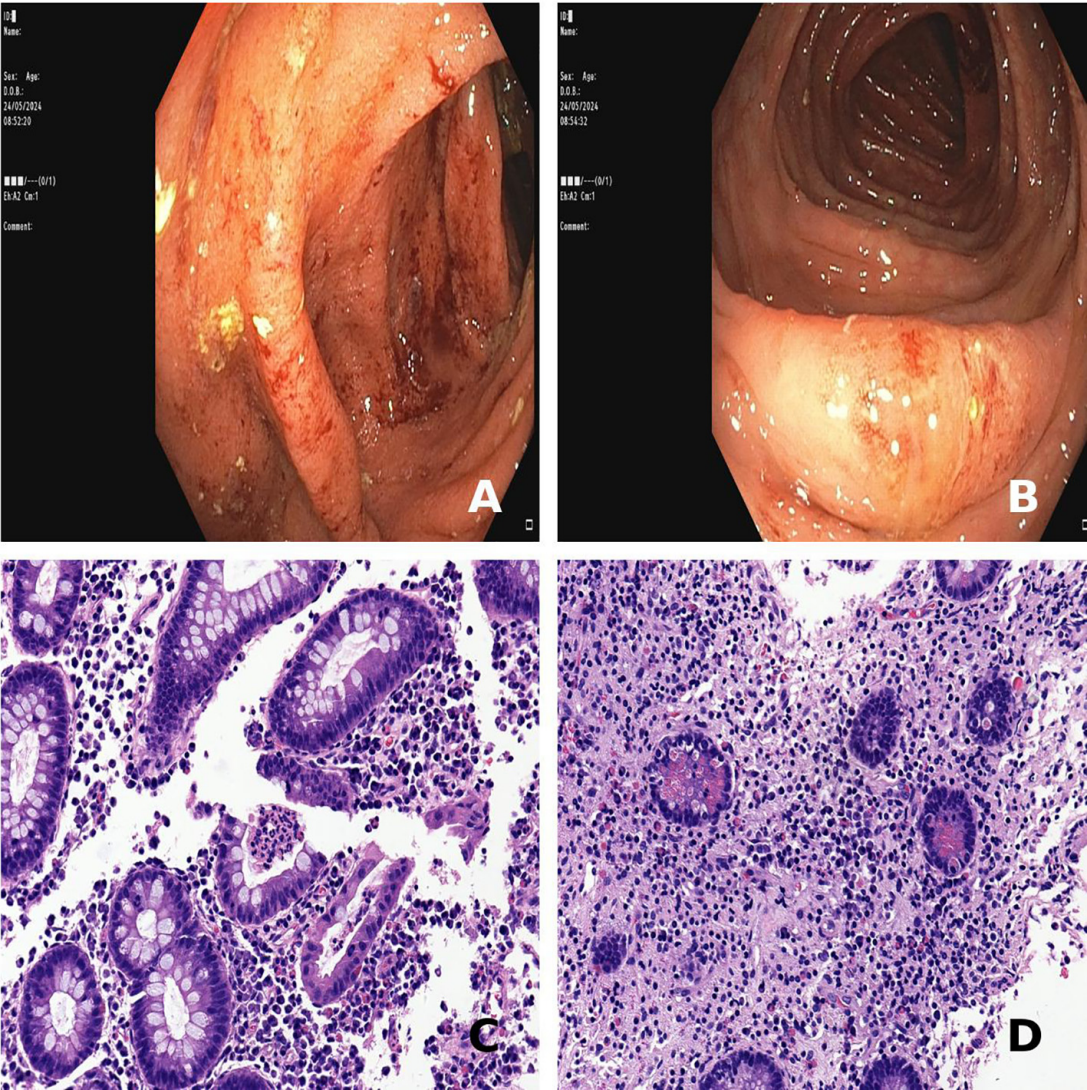
Findings of colonoscopy and colon histology consistent with Crohn’s disease. **(A, B)** Friable, easily bleeding colonic mucosa with loss of the vascular pattern from sigmoid and transverse colon, respectively. **(C)** Mild crypt architectural distorsion and active inflammation with crypt abscesses at the ascending colon (hematoxylin-eosin staining, 40x magnification). **(D)** Crypt atrophy and Paneth cell metaplasia at the descending colon (hematoxylin-eosin staining, 40x magnification).

One year later, up on corticosteroid therapy reduced to 10 mg, the patient began to suffer from gastrointestinal symptoms, such as diffuse abdominal pain and mild chronic diarrhea (up to 5 bowel movements, with signs of hematic loss). Whereas biochemistry and MRI (**Figure 1F**) ruled out the hypothesis of AIP relapse, a significant increase in calprotectin levels (607 ug/g) was found. A colonoscopy was then performed, revealing an erythematous, friable, easily bleeding mucosa with segmental involvement (**Figures 2A, B**). Histological examination showed mild crypt distortion, Paneth cell metaplasia and active inflammation of the lamina propria with multiple foci of cryptitis and crypt abscesses, with intervening areas of normal mucosa (**Figures 2C, D**), both consistent with Crohn’s disease. The association of pancreas involvement with later diagnosis of Crohn’s disease confirmed the initial hypothesis of type 2 AIP.

Two months after the onset of gastrointestinal symptoms, the patient presented with severe asthenia and scleral sub icterus. Blood tests revealed macrocytic anemia (Hb 77 g/L, MCV 109.7 fL) and indirect hyperbilirubinemia (total 73.4 µmol/L, indirect 62.3 µmol/L), with non-measurable haptoglobin and high levels of lactate dehydrogenase (585 U/L, reference range 0 – 224 U/L), to support diagnosis of hemolytic anemia. Of note, a positive direct Coombs test, showing IgG-type autoantibodies adhering to erythrocytes with complement fixation (C3d), addressed the autoimmune origin of hemolytic anemia (autoimmune hemolytic anemia, AHIA). Turning again to high-dose i.v. corticosteroids (1 mg/kg for a week), we obtained remission of sub icterus with stabilization of hemoglobin levels (106 g/L two weeks later), which then increased during the hematological follow-up keeping oral prednisone at 60 mg/day (144 g/L two months later).

## Next directions

The patient, for the moment, did not start any specific treatment for Crohn’s disease, due to the slow steroid tapering handled by the hematologist. A specific treatment for Crohn’s disease will be considered upon completion of the ongoing steroid therapy, while monitoring hemoglobin, hemolysis biomarkers and pancreatic function tests. If evidence of AIP or AIHA relapse, low-dose corticosteroids (e.g., 10 mg) will be opted. In addition to colonoscopy, an upper endoscopy is also planned in order to evaluate extension of gastrointestinal involvement of Crohn’s disease. Possibly, both procedures along with MR enterography will be performed with the patient on the minimal corticosteroid dosage according to the hematologist indications.

## Discussion

In this study, we described a case of painless obstructive jaundice occurring in a young adult, caused by a rare condition, the type 2 AIP (AIP-2), which was associated with other immune-mediated manifestations, such as Crohn’s disease and AHIA. Given the radiological evidence of distal biliary structuring within an enlarged pancreatic head, the first diagnostic dilemma was to rule out the feared eventuality of pancreatic cancer. In the young age, jaundice has been classically regarded as unlikely due to malignancy, though recent studies have highlighted the concept that the rates of more than a dozen cancers, including biliary tract and pancreas, have been increasing among adults under the age of 50 in the last decade, with early onset cancer disparities affecting more black people (1). Based on highly suggestive findings for AIP provided by MRI (the duct-penetrating sign, halo sign and sausage shape as shown in **Figure 1**), we might prioritize a non-invasive approach, consisting of corticosteroid treatment (2). This led to a fast and complete clinical response just within the first week, avoiding unnecessary and risky interventions, such as pancreas biopsy, ERCP or eventually, duodenopancreatectomy, which in the setting of biliary obstruction, can be complicated by bleeding and infections (3, 4). Even if the definitive diagnosis of AIP-2 is histological (5), performing a pancreas biopsy would have exposed the patient to an unnecessary risk given the clarity of the clinical picture, which would have benefit from corticosteroids in either AIP subtypes. From a clinical point of view, this decision was fundamental, as in the management of patients with biliary strictures, the main objective is twofold: establishing a definitive diagnosis and restoring biliary patency. In this respect, corticosteroid therapy serves as a tween-win strategy. On the one side, it acts as a therapeutic intervention, and on the other hand, it provides the *ex-adjuvantibus* criterion to support the autoimmune etiology. Noteworthy, a rapid clinical and biochemical improvement following steroid administration was almost pathognomonic for AIP, which response rates approach 100%, without the need of biliary stent placement (6). AIP-2 has been described as presenting with jaundice in approximately 25-40% of cases, less commonly than in AIP-1 (7). Unlike type 1, AIP-2 is not associated with elevated serum IgG4 levels, and typically affects younger patients. AIP-1 is increasingly recognized in subjects who present with sclerosing cholangitis, possibly representing 10% of PSC patients, with evidence of long strictures, pre-stenotic bile duct dilation, often accompanied by ‘skip’ lesion (8). Compared to AIP-2, which is more often confined to the pancreas, in AIP-1 the diagnostic work-up needs evaluation of other extra-pancreatic manifestations, in particular renal and lymph node involvement. Overall, deep investigation deserving consideration for AIP is crucial in differential diagnosis of young individuals with jaundice. This is of paramount importance, as in this age group, primary cholangiopathies are epidemiologically more relevant than in others, but their recognition may require complex processes, with repeated investigations, which not always lead to a definite diagnosis after months or even years.

Although systemic involvement is an important discriminant feature between AIP-1 and AIP-2, AIP-2 has a significant association with IBD, which was present also in our case. Studies have shown concomitant IBD in up to 30-50% of patients with AIP-2, mostly ulcerative colitis (nearly 60%) (9). Interestingly, the damage of the pancreatic duct is characterized by marked neutrophil infiltration, known as granulocytic epithelial lesions, which resembles crypt abscesses typical of inflammatory bowel disease. Of note, increased calprotectin levels were found in our case since the first admission because of jaundice, but they rapidly decreased upon steroid treatment. Nevertheless, a diagnosis of Crohn’s disease was made only one year later after reduction of prednisone dose, when the patient complaint of abdominal pain and chronic diarrhea requiring colonoscopy. Our diagnosis of Crohn’s disease, although endoscopic appearance was likely influenced by the ongoing steroid treatment, was supported by the segmental distribution of colonic lesions and the histological findings. Previous studies reported an increased severity of IBD when associated to AIP, showing a more inflammatory phenotype, less perianal involvement, and more colectomies in patients with AIP and CD than controls (10), but not an increased cancer risk (11), which instead is documented in IgG4-related sclerosing cholangitis (12). The clinical picture was further complicated, two months after the diagnosis of Crohn’s disease, by the appearance of scleral jaundice, notwithstanding the patient was on low-dose corticosteroids. However, the likelihood of AIP recurrence was ruled out by the normality of pancreatic enzymes, whereas the pre-hepatic origin of this second episode of jaundice was supported by the diagnosis of AHIA. The development of AHIA over the course of AIP-2 adds to the uniqueness of this case. Although AHIA has been documented in a few autoimmune conditions, it is a very rare complication of AIP, as in literature, only few cases of AHIA related to AIP have been reported (13, 14). Overall, our patient showed a cluster of immune-mediated conditions, encompassing AIP-2, Crohn’s disease and AHIA, all favorably responding to corticosteroids. This sequence is paradigmatic of the importance to explore alternative diagnoses, such as hemolysis, when jaundice recurs, especially in the context of autoimmune diseases.

## Conclusion

This case highlights the complex management of painless obstructive jaundice in the young, where the prospect of malignancy should, however, not be overlooked. Starting from a highly informative MRI, we went through a triple rarity: first AIP-2, which is rarer than AIP-1, second the AIP-2 association with Crohn’s disease, which is rarer than with ulcerative colitis, and third, the AIP-2 association with AHIA, which is anecdotal. In this evolving sequence of rare conditions, the decision-making process was speeded-up by the rapid response to corticosteroids, which enabled us to avoid unessential invasive procedures. Thus, in the clinical management of biliary tract diseases even if presenting with feared manifestations, it is important to be aware of such rare conditions when treatable.

## Data Availability

The raw data supporting the conclusions of this article will be made available by the authors, without undue reservation.
